# Association between physical activity and risk of renal function decline and mortality in community-dwelling older adults: a nationwide population-based cohort study

**DOI:** 10.1186/s12877-022-03693-1

**Published:** 2022-12-17

**Authors:** Hyunsuk Kim, Mun Jung Ko, Chi-Yeon Lim, Eunjin Bae, Young Youl Hyun, Sungjin Chung, Soon Hyo Kwon, Jang-Hee Cho, Kyung Don Yoo, Woo Yeong Park, In O Sun, Byung Chul Yu, Gang-Jee Ko, Jae Won Yang, Won Min Hwang, Sang Heon Song, Sung Joon Shin, Yu Ah Hong

**Affiliations:** 1 Division of Nephrology, Department of Internal Medicine, Hallym University Medical Center, Chuncheon Sacred Heart Hospital, Chuncheon, Republic of Korea; 2 Department of Biostatistics, Dongguk University College of Medicine, Goyang-Si, Republic of Korea; 3 Division of Nephrology, Department of Internal Medicine, Gyeongsang National University College of Medicine, Jinju, Republic of Korea; 4 Division of Nephrology, Department of Internal Medicine, Kangbuk Samsung Hospital, Sungkyunkwan University, School of Medicine, Seoul, Republic of Korea; 5 Division of Nephrology, Department of Internal Medicine, Yeouido St. Mary’s Hospital, College of Medicine, The Catholic University of Korea, Seoul, Republic of Korea; 6 Division of Nephrology, Department of Internal Medicine, Soonchunhyang University Seoul Hospital, Seoul, Republic of Korea; 7 Division of Nephrology, Department of Internal Medicine, Kyungpook National University Hospital, School of Medicine, Kyungpook National University, Daegu, Republic of Korea; 8 Division of Nephrology, Department of Internal Medicine, Ulsan University Hospital, University of Ulsan College of Medicine, Ulsan, Republic of Korea; 9 Division of Nephrology, Department of Internal Medicine, Keimyung University Dongsan Hospital, Keimyung University School of Medicine, Daegu, Republic of Korea; 10 Division of Nephrology, Department of Internal Medicine, Presbyterian Medical Center, Jeonju, Republic of Korea; 11 Division of Nephrology, Department of Internal Medicine, Soonchunhyang University Bucheon Hospital, Bucheon, Republic of Korea; 12 Division of Nephrology, Department of Internal Medicine, Korea University Guro Hospital, Korea University College of Medicine, Seoul, Republic of Korea; 13 Division of Nephrology, Department of Internal Medicine, Yonsei University Wonju College of Medicine, Wonju, Republic of Korea; 14 Division of Nephrology, Department of Internal Medicine, Konyang University Hospital, Daejeon, Republic of Korea; 15 Division of Nephrology, Department of Internal Medicine and Biomedical Research Institute, Pusan National University Hospital, Busan, Republic of Korea; 16 Division of Nephrology, Department of Internal Medicine, Dongguk University Ilsan Hospital, Dongguk University School of Medicine, Goyang, Republic of Korea; 17 Division of Nephrology, Department of Internal Medicine, Daejeon St. Mary’s Hospital, College of Medicine, The Catholic University of Korea, #64, Daeheung-Ro, Jung-Gu, Daejeon, 34943 Republic of Korea

**Keywords:** Physical activity, Renal function, Mortality, Older adults

## Abstract

**Background:**

Physical activity (PA) is an important risk factor associated with health outcomes. However, the relationship between PA and kidney function decline in older adults remains unclear. We examined the influence of PA on kidney function decline and mortality in community-dwelling older adults.

**Methods:**

Adults aged ≥ 65 years with an estimated glomerular filtration rate (eGFR) > 60 mL/min/1.73 m^2^ who had available health checkup data from 2009 to 2010 were included. The cohort was followed annually through December 2015 for anthropometric, sociodemographic, and medical information including outcomes and biennially for laboratory information from the health checkup. We divided these patients into three groups according to self-reported PA (Inactive group: no leisure-time PA, Active group: vigorous activity for at least 80 min/week or a sum of moderate-intensity activity and walking for at least 300 min/week, Low-active group: level of PA between the definitions of the other two groups). Associations between the intensity of PA and death, cardiovascular death, and ≥ 50% eGFR decline were investigated.

**Results:**

Among 102,353 subjects, 32,984 (32.23%), 54,267 (53.02%), and 15,102 (14.75%) were classified into the inactive, low-active, and active groups, respectively. The active group was younger, contained a higher proportion of men, and had higher frequencies of hypertension, diabetes mellitus, drinking, and smoking than the other groups. The active group had significantly lower incidence rates of mortality, cardiovascular mortality, and kidney function decline than the other groups (all *p* < 0.001). The active group also showed lower all-cause (hazard ratio [HR], 0.76; 95% confidence interval [CI], 0.70–0.82) and cardiovascular mortality (HR, 0.64; 95% CI, 0.53–0.78) and protection against ≥ 50% eGFR decline (HR, 0.81; 95% CI, 0.68–0.97) compared with the inactive group in the fully adjusted Cox proportional hazards regression model.

**Conclusions:**

High PA was an independent modifiable lifestyle factor for reducing mortality and protecting against declines in kidney function in older adults.

## Background

Longer life expectancies and low birth rates have led to substantial increase in the proportion of older people worldwide. Given that aging is a well-established risk factor for various chronic diseases [1], it is not surprising that the prevalence of chronic kidney disease (CKD) is increasing, particularly in developed countries with a growing aged population [2]. Because CKD in older people is closely associated with increased risk of cardiovascular disease, cognitive dysfunction, and functional impairment, the increasing prevalence of CKD is a substantial economic, social, and medical concern [3].

Aging is associated with sarcopenia, progressive decline in muscle mass and strength that leads to frailty [4]. Frailty reduces physiologic reserves, increases vulnerability upon exposure to stressors, and is closely related to harmful outcomes such as falls, functional impairment, cognitive dysfunction, and cardiovascular comorbidity and death in older adults [5]. Increased physical activity (PA) has been regarded as a potential strategy for mitigating sarcopenia in older adults [6]. Sarcopenia and frailty are prevalent in both older population and CKD patients and strongly associated with higher risk of hospitalization and all-cause mortality in these patients [7]. Low PA is also related to poor clinical outcomes and reduced quality of life in patients with CKD, including worsening kidney function [8–12]. Some previous studies suggested that an increase in PA reduces the risk of decline in estimated glomerular filtration rate (eGFR) and development of albuminuria in patients with CKD or general population [13–15]. However, the impact of PA on worsening kidney function remains unclear in older people.

We hypothesized that high PA is associated with reduced mortality and slow progression of kidney function decline in community-dwelling older adults who were not diagnosed as CKD and investigated that possibility using the Senior Cohort Database of the National Health Insurance Service (NHIS-Senior cohort) in Korea.

## Methods

### Data source and study participants

This nationwide, retrospective, observational study was performed using data from the NHIS-Senior cohort between 2009 and 2015. The NHIS-Senior cohort was established to support research about older people in Korea [16]. The research database contains de-identified data from approximately 558,147 individuals aged ≥ 60 years who were eligible for National Health Insurance and Medical Aid as of the end of December 2002. The cohort was followed annually through December 2015 for anthropometric, sociodemographic, and medical information including outcomes and biennially for laboratory information from the health checkup. Because serum creatinine level was added to the health checkup in 2009, study participants who underwent baseline health checkups between 2009 and 2010 were initially screened for enrollment.

**Fig. 1:**
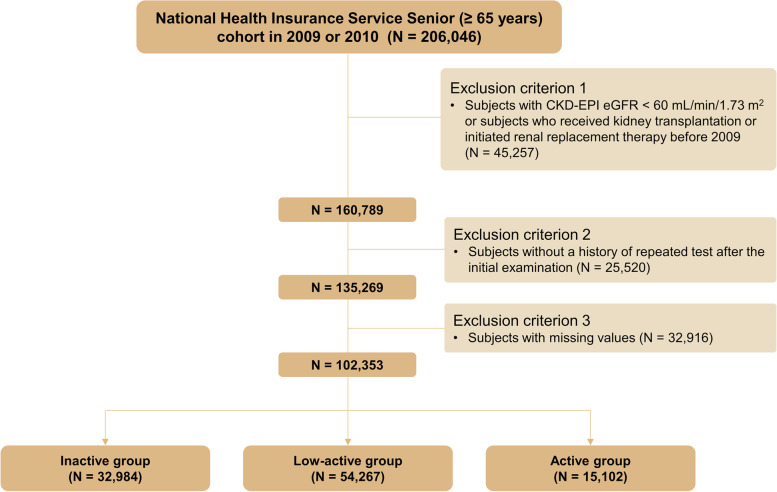
Study design and flow chart of study participants

We initially recruited 206,046 individuals aged ≥ 65 years who underwent an initial health checkup between 2009 and 2010. We excluded individuals who had baseline eGFR < 60 mL/min/1.73m^2^ or previously had received a kidney transplant or dialysis (*n* = 45,247). We also excluded individuals without follow-up data of serum creatinine level after the initial visit (*n* = 25,520) and those with missing values in the PA questionnaire (*n* = 32,916). Therefore, 102,353 individuals were included in our final analysis and followed until 2015 (Fig. 1).

### Classification of physical activity

The intensity and frequency of PA were assessed using a self-reported questionnaire, the Korean version of the International Physical Activity Questionnaire (IPAQ)-short form [17]. The IPAQ-short form asks about the frequency and duration of walking, moderate-intensity, and vigorous-intensity PA in the preceding 7 days. The World Health Organization (WHO) Guidelines recommend that older adults engage in at least 150 min/week of moderate-intensity aerobic activity, 75 min/week of vigorous-intensity aerobic activity, or an equivalent combination [18]. However, it is difficult for most older adults to achieve those targets because of age-related limitations, such as comorbidities, mobility problems, or increased risk of injury. Although the current guidelines do not include a recommendation for light-intensity PA, a recent study found that participating in at least 300 min/week of light-intensity PA was associated with favorable health outcomes in older adults [19]. Therefore, we categorized subjects into the inactive, low-active, and active groups according to reported intensity of PA, including light-intensity PA. The inactive group contained those who said that they did not engage in any PA. The active group was defined as those who performed vigorous activity for at least 80 min/week or the sum of moderate-intensity activity and walking for at least 300 min/week. The low-active group was defined as those whose PA fell between the definitions of the other two groups.

### Baseline data collection

Baseline demographic and clinical data were analyzed for all participants. Blood samples were obtained after ≥ 8-h fasting state and serum creatinine level was measured using an isotope dilution mass spectrometry–calibrated method, and eGFR was calculated using the creatinine-based CKD Epidemiology Collaboration equation [20]. Deciles of the National Health Insurance premium were investigated as a proxy measure for precise income and re-categorized into five groups.

### Outcomes

The primary outcomes were death, cardiovascular death, and a decline in eGFR within the observation period. Deaths and causes of death were ascertained from records linked with the Korean Statistical Information Service using unique personal identification numbers [21]. Cardiovascular death was defined using the following codes from the Korean Classification of Diseases version 5 or 6: E10–E14, I10–I15. I20–I25, I60–I69, I70–I79. A decline in eGFR was defined as ≥ 50% eGFR decrease from baseline [22, 23]. The change in eGFR was assessed using the baseline eGFR and the last eGFR of health checkup during the follow-up period, and the percent change in eGFR was calculated as follows: (last eGFR – baseline eGFR)/(baseline eGFR) × 100%.

### Statistical analysis

Continuous variables are expressed as mean and standard deviation, and categorical variables are presented as frequency with percentage. To test whether the variables are normally distributed, the Kolmogorov–Smirnov test was used. *P* values were obtained from Chi-square testing for categorical variables. Survival curves according to level of PA were estimated using the Kaplan–Meier method, and the significance of the survival curve was assessed by log rank testing. Hazard ratios (HRs) with 95% confidence intervals (CIs) were obtained from Cox proportional hazards regression analysis. Univariable analyses using Cox proportional hazards regression analysis were performed to determine the risk factors for death, cardiovascular death, and decline in eGFR during follow up and were followed by multivariable analyses to determine significant factors. A *p* for trend in the Cox proportional hazards model was calculated using the Wald test. Subgroup analyses were conducted using age, sex, body mass index (BMI), smoking, drinking, income, and various comorbidities. The linearity assumption for continuous variables was verified. The proportional hazards assumption for categorical variables was verified using a log-minus-log plot. All analyses were performed using R software 3.3.3 and SAS version 9.4 (SAS Institute Inc., Cary, NC, USA). *P* values < 0.05 were considered to indicate statistical significance.

## Results

### Baseline characteristics

Table 1 shows the baseline characteristics of the study population by PA level. The mean age of the total population was 72.0 ± 4.0 years, and 46,183 subjects (45.12%) were male. The subjects were categorized into the inactive group (*n* = 32,984, 32.23%), low-active group (*n* = 54,267, 53.02%), and active group (*n* = 15,102, 14.75%). The active group was relatively young, containing many 65- to 74-year-old subjects. The subjects in the active group were more likely to be men (61.28%) compared with the inactive and low-active groups. Interestingly, the participants in the active group were more likely than the others to be ex-smokers (23.16%) and alcohol consumers (34.66%) and less likely to be non-smokers (65.69%). The proportion of current smokers was similar among the three groups. The active group also had higher prevalence of diabetes mellitus (20.29%), hypertension (54.85%), and dyslipidemia (7.4%) than the other groups. Baseline eGFR level did not differ among the groups. The proportion of very high decile of income was relatively high in the active group (42.52%).

**Table 1:** Baseline characteristics of the subjects according to the levels of physical activity in older adults

		Inactive	Low-active	Active
Number, *n* (%)		32,984 (32.23)	54,267 (53.02)	15,102 (14.75)
Age (year)		72.4 ± 4.2	71.6 ± 3.9	71.4 ± 3.6
Age group (year, *n* (%))	65–74	24,730 (74.98)	43,182 (79.57)	12,746 (84.4)
	75–84	7,879 (23.89)	10,748 (19.81)	2,296 (15.2)
	85-	375 (1.14)	337 (0.62)	60 (0.4)
Sex (male, *n* (%))		12,616 (38.25)	24,312 (44.8)	9,255 (61.28)
BMI (kg/m^2^, *n* (%))	< 18.5	1,289 (3.91)	1,726 (3.18)	331 (2.19)
	18.5–24.9	20,027 (60.72)	33,120 (61.03)	9,437 (62.49)
	≥ 25	11,668 (35.37)	1,9421 (35.79)	5,334 (35.32)
BMI (kg/m^2^)		23.95 ± 3.27	24.01 ± 3.07	24.02 ± 2.86
SBP (mmHg)		131.38 ± 16.56	130.45 ± 15.76	130.37 ± 15.38
Smoking, *n* (%)	Non-smoker	25,883 (78.47)	40,105 (73.9)	9,920 (65.69)
	Ex-smoker	3,288 (9.97)	8,367 (15.42)	3,498 (23.16)
	Current smoker	3,813 (11.56)	5,795 (10.68)	1,684 (11.15)
Alcohol, *n* (%)		6,541 (19.83)	13,749 (25.34)	5,234 (34.66)
CVD, *n* (%)		1,124 (3.41)	1,797 (3.31)	507 (3.36)
Heart disease, *n* (%)		2,546 (7.72)	4,417 (8.14)	1,205 (7.98)
Diabetes mellitus, *n* (%)		5,030 (15.25)	9,300 (17.14)	3,064 (20.29)
Hypertension, *n* (%)		16,963 (51.43)	29,302 (54)	8,284 (54.85)
Dyslipidemia, *n* (%)		1,604 (4.86)	3,662 (6.75)	1,117 (7.4)
Creatinine (mg/dL)		0.82 ± 0.17	0.84 ± 0.17	0.88 ± 0.17
eGFR (mL/min /1.73 m^2^)		79.08 ± 11.71	78.87 ± 11.57	78.68 ± 11.48
Fasting glucose (mg/dL)		102.74 ± 25.98	103.7 ± 26.14	105.5 ± 26.82
Total cholesterol (mg/dL)		197.19 ± 39.13	195.76 ± 38.29	192.95 ± 37.66
Income, *n* (%)	0–2	5,314 (16.11)	8,269 (15.24)	2,175 (14.4)
	3–4	4,136 (12.54)	6,248 (11.51)	1,539 (10.19)
	5–6	5,113 (15.5)	7,417 (13.67)	1,976 (13.08)
	7–8	6,696 (20.3)	10,633 (19.59)	2,990 (19.8)
	9–10	11,725 (35.55)	21,700 (39.99)	6,422 (42.52)

*Abbreviations: BMI* Body mass index, *CVD* Cerebrovascular disease, *DBP* Diastolic blood pressure, *eGFR* estimated glomerular filtration rate, *HDL-C* High-density lipoprotein cholesterol, *LDL-C* Low-density lipoprotein cholesterol, *SBP* Systolic blood pressure

### Association between physical activity and risks of deaths and renal function decline

**Fig. 2:**
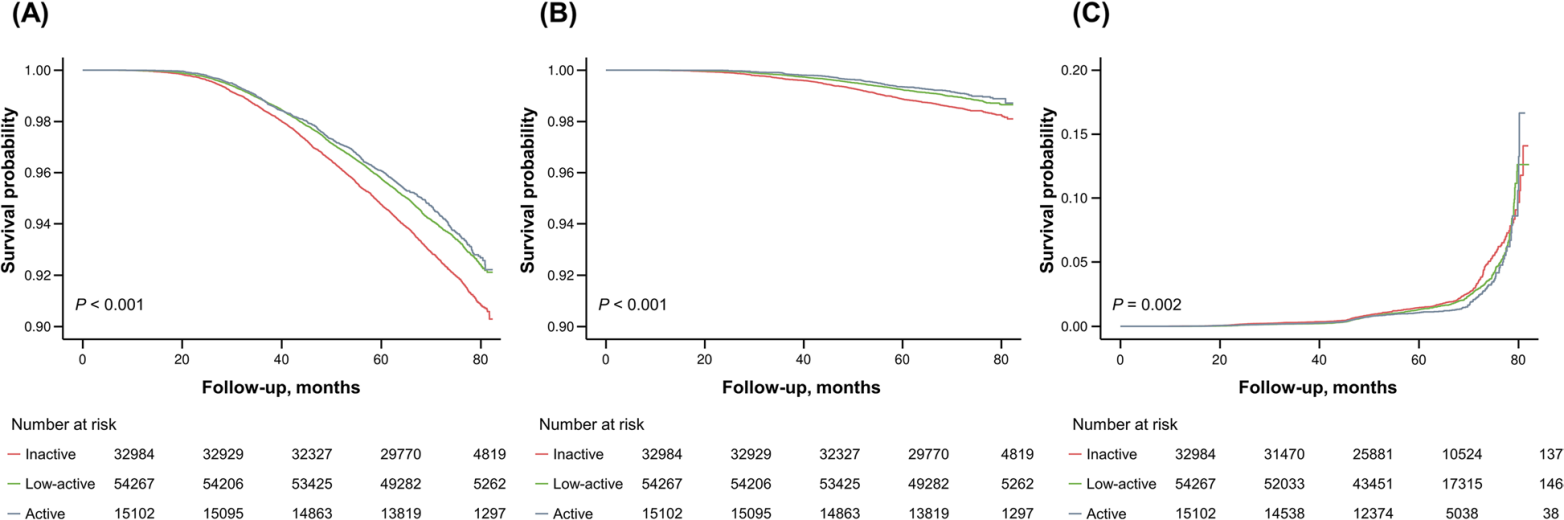
Kaplan–Meier survival curve for (**A**) all-cause, (**B**) cardiovascular mortality and (**C**) ≥ 50% eGFR decline according to the levels of physical activity in older adults

During a mean follow-up period of 69.8 ± 9.0 months (range, 6.9–82.4 months), 6,594 (6.44%) subjects died, and 1,345 (1.31%) subjects experienced a greater than 50% decrease in renal function (Table 2). Overall, the incidence rates of all-cause death and cardiovascular death per 1000 person-years were 12.57 and 2.45, respectively, in the inactive group; 10.18 and 1.74 in the low-active group; and 9.55 and 1.48 in the active group. The incidence rate of decline in eGFR was 3.44 in the inactive group, 2.92 in the low-active group, and 2.59 in the active group. The active group had significantly lower incidence rates of mortality, cardiovascular mortality, and renal function decline than the other groups (all *p* < 0.001). The inactive group had poorer survival than the low-active and active groups (both *p* < 0.001 by log rank, Fig. 2A and B). The cumulative incidence of renal function decline was higher in the inactive group than in the low-active and active groups (*p* = 0.002, by log rank, Fig. 2C).

**Table 2:** Mortality and renal events according to the levels of physical activity in older adults

	Observed (*n*)	Events, *n* (%)	Person-years	Incidence rates /1,000 person-years	*p* value
**All-cause mortality**					< 0.001
Inactive	32,984	2,470 (7.49)	196,482.95	12.57	
Low-active	54,267	3,268 (6.02)	321,152.13	10.18	
Active	15,102	8,54 (5.65)	89,379.36	9.55	
**Cardiovascular mortality**					< 0.001
Inactive	32,984	482 (1.46)	196,482.95	2.45	
Low-active	54,267	560 (1.03)	321,152.13	1.74	
Active	15,102	132 (0.87)	89,379.36	1.48	
** ≥ 50% eGFR decline**					< 0.001
Inactive	32,984	487 (1.48)	141,650.55	3.44	
Low-active	54,267	686 (1.26)	234,851.68	2.92	
Active	15,102	172 (1.14)	66,356.70	2.59	

*Analysis:* A *p* value obtained from the Chi-square test

Next, we analyzed the relative risk of mortality and renal function decline during follow up according to PA level to determine the relationships between clinical outcomes and intensity of PA in older people (Table 3). The inactive group was used as the reference category for calculating HRs. In the crude model, the HRs for all-cause and cardiovascular mortality were 0.82 (95% CI, 0.78–0.86) and 0.72 (95% CI, 0.63–0.81), respectively, for the low-active group and 0.77 (95% CI, 0.71–0.83) and 0.61 (95% CI, 0.50–0.74) for the active group. Consistent with mortality rates, the HRs for renal function decline in the crude model of the low-active (HR, 0.87; 95% CI, 0.77–0.97) and active groups (HR, 0.75; 95% CI, 0.63–0.89) were significantly lower than in the inactive group. In models 1 and 2 of the multivariable analysis, the active group had significantly lower risk for all-cause mortality, cardiovascular mortality, and renal function decline than did the inactive group. In model 3, the fully adjusted Cox model that controlled for age, sex, comorbidities, income, SBP, and baseline biochemical characteristics, the active group had a 25% risk reduction for all-cause mortality (HR, 0.76; 95% CI 0.70–0.82) and an about 35% risk reduction for cardiovascular mortality (HR, 0.64; 95% CI 0.53–0.78). Similar results were observed in the association between the low-active group and all-cause mortality (HR 0.84, 95% CI 0.80–0.89) and cardiovascular mortality (HR 0.75, 95% CI 0.67–0.85). The active group remained associated with a significantly lower risk of renal function decline in the fully adjusted Cox regression model (HR 0.81, 95% CI 0.68–0.97), but the low-active group did not. The *p* for trends across the three groups were all significant indicating that the higher PA, the lower all-cause mortality, cardiovascular mortality, and renal function decline.

**Table 3:** Associations between the levels of physical activity and clinical outcomes in the elderly

	Crude			Model 1			Model 2			Model 3		
	HR	95% CI		HR	95% CI		HR	95% CI		HR	95% CI	
		Lower	Upper		Lower	Upper		Lower	Upper		Lower	Upper
**All-cause mortality**												
Inactive (Ref.)	1			1			1			1		
Low-active	0.82	0.78	0.86	0.83	0.79	0.87	0.84	0.80	0.89	0.84	0.80	0.89
Active	0.77	0.71	0.83	0.73	0.68	0.79	0.76	0.70	0.82	0.76	0.70	0.82
*p* for trend	< 0.001			< 0.001			< 0.001			< 0.001		
**Cardiovascular mortality**												
Inactive (Ref.)	1			1			1			1		
Low-active	0.72	0.63	0.81	0.75	0.66	0.85	0.75	0.66	0.85	0.75	0.67	0.85
Active	0.61	0.50	0.74	0.63	0.52	0.77	0.64	0.53	0.78	0.64	0.53	0.78
*p* for trend	< 0.001			< 0.001			< 0.001			< 0.001		
** ≥ 50% eGFR decline**												
Inactive (Ref.)	1			1			1			1		
Low-active	0.87	0.77	0.97	0.90	0.80	1.03	0.88	0.78	0.99	0.91	0.81	1.03
Active	0.75	0.63	0.89	0.82	0.68	0.97	0.78	0.65	0.93	0.81	0.68	0.97
*p* for trend	0.001			0.014			0.004			0.021		

*Analysis:* A *p* for trend obtained from the Wald test, and hazard ratios with 95% confidence intervals were obtained from Cox proportional hazards regression analysis

Model 1: age, sex, body mass index, Model 2: Model 1 + smoking, alcohol, income, cerebrovascular disease, heart disease, diabetes mellitus, hypertension, dyslipidemia, Model 3: Model 2 + systolic blood pressure, fasting glucose, total cholesterol, baseline estimated glomerular filtration rate

### Subgroup analyses of the risks of deaths and renal function decline

**Fig. 3:**
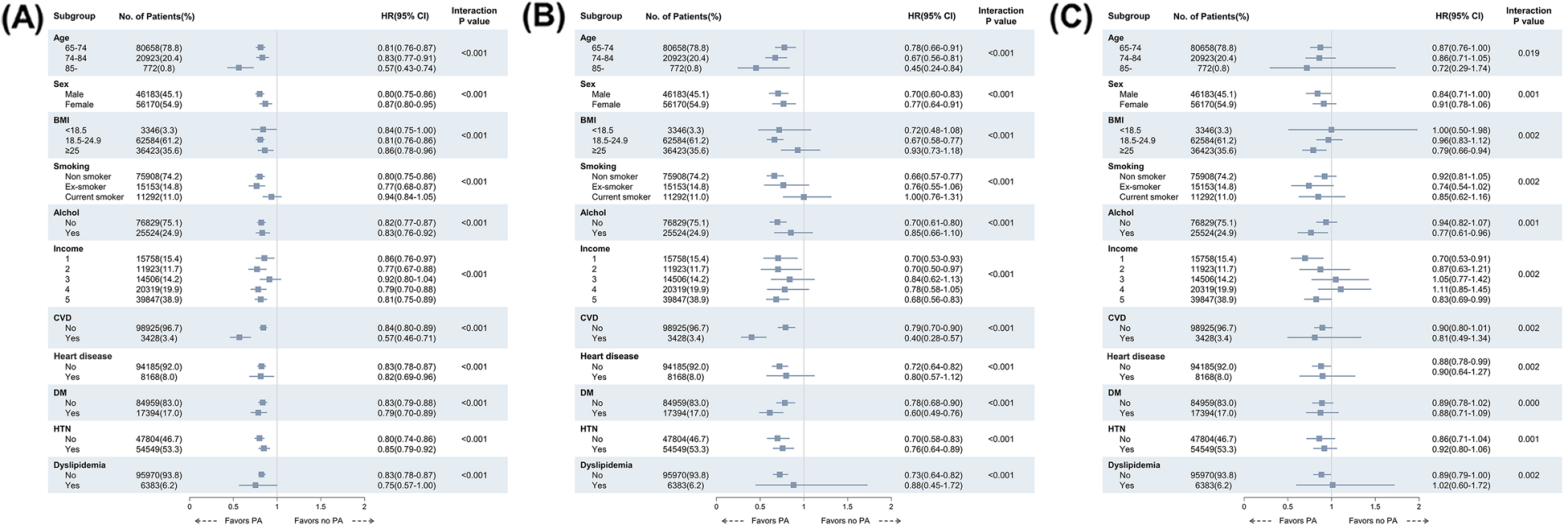
Subgroup analysis for (**A**) all-cause mortality, (**B**) cardiovascular mortality and (**C**) ≥ 50% eGFR decline in the inactive vs. the low or active groups of physical activity in subgroups of older adults. BMI, body mass index; CI, confidence interval; CVD, cerebrovascular disease; DM, diabetes mellitus; HD, heart disease; HR, hazard ratio; HTN, hypertension; No, number; PA, physical activity. Adjusted for age, sex, body mass index, smoking, alcohol, income, cerebrovascular disease, heart disease, hypertension, dyslipidemia, systolic blood pressure, fasting glucose, total cholesterol, and baseline estimated glomerular filtration rate

To elucidate the effects of subgroups on the associations between PA and clinical outcomes, we performed subgroup analyses stratified by age, sex, BMI, smoking, alcohol, income, and comorbidities. Subgroup analyses were performed after adjustments for age, sex, BMI, smoking, alcohol, income, cerebrovascular disease, heart disease, diabetes mellitus hypertension, dyslipidemia, SBP, fasting glucose, total cholesterol, and baseline eGFR. The effect of PA on all-cause mortality substantially prominent in subjects 85 years and older (HR 0.57, 95% CI 0.43–0.74) and subjects with cerebrovascular disease (HR 0.57, 95% CI 0.46–0.71). PA was found to be beneficial regardless of sex, alcohol consumption, and the presence of cerebrovascular disease, heart disease, diabetes mellitus, or hypertension. In the case of current smokers (HR 0.94, 95% CI 0.84–1.05) and underweight (BMI < 18.5 kg/m^2^, HR 0.84, 95% CI 0.75–1.00), there was no beneficial effect of PA on all-cause mortality (Fig. 3A).

The positive effect of PA on cardiovascular death was found regardless of age, sex, and the presence of cerebrovascular disease, diabetes mellitus, or hypertension. However, in subgroup analyses according to BMI and smoking status, the effect of PA was limited only in the subjects with BMI 18.5–24.9 kg/m^2^ (HR 0.67, 95% CI 0.58–0.77) or non-smokers (HR 0.66, 95% CI 0.57–0.77). In those with heart disease, PA reduced all-cause mortality but had no significant influence on cardiovascular mortality (HR 0.80, 95% CI 0.57–1.12). Consistently, PA had no significant influence on cardiovascular mortality in the subgroup with dyslipidemia (HR 0.88, 95% CI 0.45–1.72, Fig. 3B).

The association between PA and more than 50% decline in eGFR became unclear when the subjects were divided by age. A significant effect of PA was found only in the group with BMI ≥ 25 kg/m^2^ (HR 0.79, 95% CI 0.66–0.94). In addition, the HR of kidney function decline in the PA group was significantly lower in alcohol consumers (HR 0.77, 95% CI 0.61–0.96) and the very low- and very high-income subgroups (very low: HR 0.70, 95% CI 0.53–0.91, very high: HR 0.83, 95% CI 0.69–0.99). However, the effects of sex, smoking or variable comorbidities on kidney function decline were not clear (Fig. 3C).

## Discussion

This nationwide observational study demonstrates that any level of PA significantly lowers the risk of all-cause and cardiovascular mortality among community-dwelling subjects aged ≥ 65 years in Korea, compared with those who are physically inactive. We also confirmed that high PA was independently associated with prevention of renal function decline in older adults. In our study population, higher PA was dose-dependently associated with lower all-cause and cardiovascular mortality and renal function decline during follow up.

The positive relationships between high PA and long-term health outcomes have been demonstrated in older population [24–28]. Nationwide studies demonstrated that older adults with high PA were associated with 0.48–0.73 lower of all-cause death compared with those with no PA [24, 25]. A Taiwan nationwide study also reported that exercise 3–5 times a week had a significantly decreased risk of mortality in older adults [26]. Regardless of the number of underlying diseases, high PA was associated with a mortality reduction of 30–47% in older adults [28]. Recently, a meta-analysis showed that the most sedentary participants had a 2.44 higher risk of all-cause mortality than the least sedentary participants, and the least active participants were associated with 3.09 higher risk of all-cause mortality compared with the most active participants for total PA in older adults [29]. We also found that high PA is associated with a 25% lower risk of all-cause mortality and a 36% lower risk of cardiovascular mortality in a community-dwelling older population.

Despite the convincing evidence of significant associations between PA and all-cause mortality, the causality within the relationship is not fully understood. Several pathophysiological mechanisms have been suggested to explain how PA delays mortality. PA protects against age-related mitochondrial fragmentation in skeletal muscle [30], reduces insulin resistance [31], modulates oxidative stress and inflammatory cytokines [32], and improves defective autophagy [33]. Clinically, PA has been identified as a potential preventive strategy to slow the development of frailty and sarcopenia in both older adults and CKD patients. Recent meta-analyses showed that PA prevents frailty or sarcopenia in adults aged ≥ 65 years [6], and regular PA improves the physical and functional capacity of CKD patients [34]. Therefore, an increase in physical capacity through appropriate PA might delay mortality by improving cardiorespiratory and metabolic fitness, regulating cardiovascular risk factors, reducing multimorbidity, and preventing frailty and disability.

The beneficial effects of PA on decline in renal function have been widely evaluated, but conflicting results have been reported about the relationship between PA and renal function. In a longitudinal cohort study, higher PA level was associated with slower rate of eGFR loss in patients with established CKD [35]. However, other researchers reported finding no association between PA level and progression to kidney failure in patients with advanced CKD [12]. They suggested that PA might not be a modifiable risk factor in preventing CKD progression in patients whose GFR was already low [12]. Among the older adults, higher level of PA was associated with a 28% lower adjusted risk of rapid decline in kidney function (decline ≥ 3.0 mL/min/1.73m^2^ per year in GFR) [36]. However, another study found that high sedentary time, not low level of PA, played an important role in incident CKD and rapidly declining kidney function in people aged 70–79 years [37]. Our results here support the beneficial effects of PA in preventing a decline in kidney function among older adults and confirm the importance of maintaining PA to renal health outcomes in the older population.

One noteworthy finding of this study is that the current recommendations for PA intensity are effective for protecting against renal function decline in older adults. Several global and national institutions now recommend that older adults participate in PA to gain health benefits, but most older adults do not meet those guidelines. One study reported that 27.3–44.3% of adults aged ≥ 65 years in the United States achieved the recommended level of PA [38]. In this study, we classified PA level based on WHO recommendations, and only 14.75% of our study population met those PA level. Although only the active group was independently associated with delay in renal function decline, even the low-active group was significantly associated with reduced all-cause and cardiovascular mortality, compared with the inactive group. This finding suggested that any level of PA is helpful to improve general health outcomes, and that the importance of encouraging increased PA, even in the slightest, should be emphasized in older adults. Further study may be needed to investigate the minimal target level of PA associated with survival and renal function decline.

Another interesting finding of this study is that PA was an independent risk factor for mortality and renal function decline after controlling for various lifestyle factors. The relationships between PA and other health-related behaviors are complex. Generally, low PA is known to be related to other unhealthy behaviors, such as overeating, heavy smoking, and excessive alcohol consumption [39]. In the present study, we observed that our active group was more likely than those in the other groups to be ex-smokers and alcohol consumers, and they already had a higher prevalence of diabetes mellitus, hypertension, and dyslipidemia. Nevertheless, high PA was significantly associated with reduced all-cause and cardiovascular mortality and delayed renal function decline after adjustment for unhealthy behaviors and multiple comorbidities. There was a tendency for significant interactions to exist between PA and all-cause death even in older adults with alcohol consumers or those with various comorbidities in the subgroup analyses. These findings suggest that appropriate PA is an important modifiable factor to improve survivals and prevent renal function decline even in older adults with various comorbidities or alcohol consumers.

Our study has several limitations. First, although the IPAQ-short form is easily performed and widely used to assess PA, the self-reported method risks errors caused by recall bias and misinterpretation of questions. Second, because the IPAQ-short form assesses PA based mainly on leisure time PA, the actual PA level could be underestimated in participants with physically demanding occupations. Third, we used creatinine-based eGFR to assess renal function, but that equation is insufficient in older adults. Serum creatinine is produced by muscle, and loss of muscle mass associated with aging reduces creatinine generation. Recent guidelines recommend using the cystatin C-based eGFR measurement for older people [40]. Therefore, further research using the cystatin C-based eGFR might be required to redeem this weakness. Lastly, this study has some limitations due to the nature of the observational, retrospective design. As observational studies cannot establish cause-effect relationships, potential residual biases from measured or unmeasured confounders may have influenced the results. Additionally, the most enrolled participants were followed for approximately 70 months in this study, but some participants were followed for relatively short follow-up periods. Therefore, the possibility of loss due to follow-up bias due to the difference in follow-up duration may be existed. Further well-designed prospective cohort studies or randomized controlled trials may be needed to elucidate a direct association between PA and renal function decline in older adults. Despite those limitations, a major strength of our study is the reliability of the data, which we obtained from a large, nationwide, population-based database containing PA data. To our knowledge, this is the first nationwide study to identify potential association between PA level and renal function decline in community-dwelling older adults. Moreover, we adjusted for potential confounding factors and conducted extensive subgroup analyses.

## Conclusions

High PA is an independent modifiable lifestyle factor for reducing mortality and protecting kidney function decline in older adults. Our findings underscore the need for interventional research to elucidate the effects of maintaining or increasing PA on age-related decline in kidney function of older people.

## Data Availability

Publicly available datasets of Korea were used in this study. These can be found in the NHIS-Senior cohort at https://nhiss.nhis.or.kr., reference number [16].

## Abbreviations

PA: Physical activity
eGFR: Estimated glomerular filtration rate
CKD: Chronic kidney disease
NHIS: National Health Insurance Service
IPAQ: International Physical Activity Questionnaire
WHO: World Health Organization
BMI: Body mass index
SBP: Systolic blood pressure
DBP: Diastolic blood pressure
HRs: Hazard ratios

## References

[1] Atella V, Piano Mortari A, Kopinska J, Belotti F, Lapi F, Cricelli C, Fontana L (2019). Trends in age-related disease burden and healthcare utilization. Aging Cell.

[2] Jha V, Garcia-Garcia G, Iseki K, Li Z, Naicker S, Plattner B, Saran R, Wang AY, Yang CW (2013). Chronic kidney disease: global dimension and perspectives. Lancet.

[3] Stevens LA, Viswanathan G, Weiner DE (2010). Chronic kidney disease and end-stage renal disease in the elderly population: current prevalence, future projections, and clinical significance. Adv Chronic Kidney Dis.

[4] Wilson D, Jackson T, Sapey E, Lord JM (2017). Frailty and sarcopenia: The potential role of an aged immune system. Ageing Res Rev.

[5] Clegg A, Young J, Iliffe S, Rikkert MO, Rockwood K (2013). Frailty in elderly people. Lancet.

[6] Oliveira JS, Pinheiro MB, Fairhall N, Walsh S, Chesterfield Franks T, Kwok W, Bauman A, Sherrington C (2020). Evidence on physical activity and the prevention of frailty and sarcopenia among older people: a systematic review to inform the world health organization physical activity guidelines. J Phys Act Health.

[7] Wong L, Duque G, McMahon LP (2021). Sarcopenia and frailty: challenges in mainstream nephrology practice. Kidney Int Rep.

[8] Tsai YC, Chen HM, Hsiao SM, Chen CS, Lin MY, Chiu YW, Hwang SJ, Kuo MC (2017). Association of physical activity with cardiovascular and renal outcomes and quality of life in chronic kidney disease. PLoS One.

[9] Chen JL, Lerner D, Ruthazer R, Castaneda-Sceppa C, Levey AS (2008). Association of physical activity with mortality in chronic kidney disease. J Nephrol.

[10] Beddhu S, Baird BC, Zitterkoph J, Neilson J, Greene T (2009). Physical activity and mortality in chronic kidney disease (NHANES III). Clin J Am Soc Nephrol.

[11] Kuo CP, Tsai MT, Lee KH, Lin YP, Huang SS, Huang CC, Tseng WC, Tarng DC. Dose-response effects of physical activity on all-cause mortality and major cardiorenal outcomes in chronic kidney disease. Eur J Prev Cardiol. 2022;29(3):452–61. 10.1093/eurjpc/zwaa162 33704426

[12] Rampersad C, Brar R, Connelly K, Komenda P, Rigatto C, Prasad B, Bohm C, Tangri N. Association of Physical Activity and Poor Health Outcomes in Patients With Advanced CKD. Am J Kidney Dis . 2021;78(3):391–98. 10.1053/j.ajkd.2020.12.018 33581165

[13] Martens RJH, van der Berg JD, Stehouwer CDA, Henry RMA, Bosma H, Dagnelie PC, van Dongen M, Eussen S, Schram MT, Sep SJS (2018). Amount and pattern of physical activity and sedentary behavior are associated with kidney function and kidney damage: The Maastricht Study. PLoS One.

[14] Parvathaneni K, Surapaneni A, Ballew SH, Palta P, Rebholz CM, Selvin E, Coresh J, Grams ME (2021). Association Between Midlife Physical Activity and Incident Kidney Disease: The Atherosclerosis Risk in Communities (ARIC) Study. Am J Kidney Dis.

[15] Robinson ES, Fisher ND, Forman JP, Curhan GC (2010). Physical activity and albuminuria. Am J Epidemiol.

[16] Kim YI, Kim YY, Yoon JL, Won CW, Ha S, Cho KD, Park BR, Bae S, Lee EJ, Park SY (2019). Cohort Profile: National health insurance service-senior (NHIS-senior) cohort in Korea. BMJ Open.

[17] Oh JY, Yang YJ, Kim BS, Kang JH (2007). Validity and Reliability of Korean Version of International Physical Activity Questionnaire (IPAQ) Short Form. J Korean Acad Fam Med.

[18] Bull FC, Al-Ansari SS, Biddle S, Borodulin K, Buman MP, Cardon G, Carty C, Chaput JP, Chastin S, Chou R (2020). World Health Organization 2020 guidelines on physical activity and sedentary behaviour. Br J Sports Med.

[19] Loprinzi PD, Lee H, Cardinal BJ (2015). Evidence to support including lifestyle light-intensity recommendations in physical activity guidelines for older adults. Am J Health Promot.

[20] Levey AS, Stevens LA (2010). Estimating GFR using the CKD Epidemiology Collaboration (CKD-EPI) creatinine equation: more accurate GFR estimates, lower CKD prevalence estimates, and better risk predictions. Am J Kidney Dis.

[21] Song SO, Jung CH, Song YD, Park CY, Kwon HS, Cha BS, Park JY, Lee KU, Ko KS, Lee BW (2014). Background and data configuration process of a nationwide population-based study using the korean national health insurance system. Diabetes Metab J.

[22] Yamanouchi M, Furuichi K, Shimizu M, Toyama T, Yamamura Y, Oshima M, Kitajima S, Hara A, Iwata Y, Sakai N (2022). Serum hemoglobin concentration and risk of renal function decline in early stages of diabetic kidney disease: a nationwide, biopsy-based cohort study. Nephrol Dial Transplant.

[23] Lee MJ, Chang TI, Lee J, Kim YH, Oh KH, Lee SW, Kim SW, Park JT, Yoo TH, Kang SW (2019). Urine Osmolality and Renal Outcome in Patients with Chronic Kidney Disease: Results from the KNOW-CKD. Kidney Blood Press Res.

[24] Llamas-Velasco S, Villarejo-Galende A, Contador I, Lora Pablos D, Hernández-Gallego J, Bermejo-Pareja F (2016). Physical activity and long-term mortality risk in older adults: A prospective population based study (NEDICES). Prev Med Rep.

[25] Shaked O, Cohen G, Goshen A, Shimony T, Shohat T, Gerber Y. Physical activity and long-term mortality risk in older adults with and without cardiovascular disease: a nationwide cohort study. Gerontology. 2022;68(5):529–37. 10.1159/000518169 34515134

[26] Lai YJ, Yen YF, Chen LJ, Ku PW, Chen CC, Lin YK. Association of exercise with all-cause mortality in older Taipei residents. Age Ageing 2020;49(3):382–88. 10.1093/ageing/afz172 31971585

[27] Higueras-Fresnillo S, Cabanas-Sánchez V, García-Esquinas E, Rodríguez-Artalejo F, Martinez-Gomez D (2018). Physical activity attenuates the impact of poor physical, mental, and social health on total and cardiovascular mortality in older adults: a population-based prospective cohort study. Qual Life Res.

[28] Martinez-Gomez D, Guallar-Castillon P, Garcia-Esquinas E, Bandinelli S, Rodríguez-Artalejo F (2017). Physical activity and the effect of multimorbidity on all-cause mortality in older adults. Mayo Clin Proc.

[29] Rojer AGM, Ramsey KA, Trappenburg MC, van Rijssen NM, Otten RHJ, Heymans MW, Pijnappels M, Meskers CGM, Maier AB (2020). Instrumented measures of sedentary behaviour and physical activity are associated with mortality in community-dwelling older adults: a systematic review, meta-analysis and meta-regression analysis. Ageing Res Rev.

[30] Halling JF, Ringholm S, Olesen J, Prats C, Pilegaard H (2017). Exercise training protects against aging-induced mitochondrial fragmentation in mouse skeletal muscle in a PGC-1α dependent manner. Exp Gerontol.

[31] Peng PS, Kao TW, Chang PK, Chen WL, Peng PJ, Wu LW (2019). Association between HOMA-IR and Frailty among U.S. Middle-aged and Elderly Population. Sci Rep.

[32] Sallam N, Laher I (2016). Exercise modulates oxidative stress and inflammation in aging and cardiovascular diseases. Oxid Med Cell Longev.

[33] Park SS, Seo YK, Kwon KS (2019). Sarcopenia targeting with autophagy mechanism by exercise. BMB Rep.

[34] Nakamura K, Sasaki T, Yamamoto S, Hayashi H, Ako S, Tanaka Y (2020). Effects of exercise on kidney and physical function in patients with non-dialysis chronic kidney disease: a systematic review and meta-analysis. Sci Rep.

[35] Robinson-Cohen C, Littman AJ, Duncan GE, Weiss NS, Sachs MC, Ruzinski J, Kundzins J, Rock D, de Boer IH, Ikizler TA (2014). Physical activity and change in estimated GFR among persons with CKD. J Am Soc Nephrol.

[36] Robinson-Cohen C, Katz R, Mozaffarian D, Dalrymple LS, de Boer I, Sarnak M, Shlipak M, Siscovick D, Kestenbaum B (2009). Physical activity and rapid decline in kidney function among older adults. Arch Intern Med.

[37] Hawkins M, Newman AB, Madero M, Patel KV, Shlipak MG, Cooper J, Johansen KL, Navaneethan SD, Shorr RI, Simonsick EM (2015). TV watching, but not physical activity, is associated with change in kidney function in older adults. J Phys Act Health.

[38] Keadle SK, McKinnon R, Graubard BI, Troiano RP (2016). Prevalence and trends in physical activity among older adults in the United States: a comparison across three national surveys. Prev Med.

[39] Blair SN, Jacobs DR, Powell KE (1985). Relationships between exercise or physical activity and other health behaviors. Public Health Rep.

[40] Farrington K, Covic A, Nistor I, Aucella F, Clyne N, De Vos L, Findlay A, Fouque D, Grodzicki T, Iyasere O (2017). Clinical Practice Guideline on management of older patients with chronic kidney disease stage 3b or higher (eGFR<45 mL/min/1.73 m2): a summary document from the European Renal Best Practice Group. Nephrol Dial Transplant.

